# The patterns of uveitis and the factors affecting visual outcome from Chulalongkorn University Uveitis Cohort (CU^2^C): A 5-year longitudinal study protocol

**DOI:** 10.1371/journal.pone.0275666

**Published:** 2022-10-06

**Authors:** Jakkrit Juhong, Krit Pongpirul, Somtaporn Ueathaweephol, Thanapong Somkijrungroj, Wijak Kongwattananon

**Affiliations:** 1 Department of Ophthalmology, Faculty of Medicine, Chulalongkorn University, Bangkok, Thailand; 2 Department of Preventive and Social Medicine, Faculty of Medicine, Chulalongkorn University, Bangkok, Thailand; 3 Department of Ophthalmology, Burachat Chaiyakorn Hospital, Bangkok, Thailand; 4 Center of Excellence in Retina, Faculty of Medicine, Chulalongkorn University, Bangkok, Thailand; Cairo University Kasr Alainy Faculty of Medicine, EGYPT

## Abstract

**Background:**

In Thailand, several novel laboratory investigations are recently available to help differentiate the uveitic etiologies. The update on uveitis epidemiological data in Thailand is necessary to better understand the disease burden and provide guidance on management. The current study aims to describe the prevalence and identify factors associated with poor visual outcomes of uveitis patients at a tertiary center in Thailand.

**Methods:**

A 5-year-prospective study of uveitis cases presented at a tertiary referral center in the central region of Thailand is conducted.

## Introduction

Uveitis is a group of intraocular inflammation affecting the uveal tract and adjacent ocular structures such as retina, optic nerve, and vitreous [1]. The classification of uveitis is generally based on the primary site of intraocular inflammation which includes anterior, intermediate, posterior, and panuveitis [2]. Various genetic, geographic, nutritional, socioeconomic, ethnic, and environmental factors are known to affect the patterns of uveitis [2, 3].

The epidemiology of uveitis varies among different parts of the world. Nearly half of all uveitis cases in developing countries are caused by toxoplasmosis or tuberculosis [4]. In contrast, idiopathic anterior uveitis and human leukocyte antigen (HLA) -B27 positive associated anterior uveitis [4] were more prevalent in developed countries [4] Also, infectious causes were found to be less common, accounting for 11–21 percent of uveitic cases. In Thailand, epidemiological data on uveitis from different regions have been reported. Cytomegaloviral retinitis (CMVR) is the most common cause of uveitis in the northern region [3], while ocular toxoplasmosis is the most frequent in the south [5]. In the central region, herpetic anterior uveitis was found to be the major uveitic cause [6].

Uncontrolled ocular inflammation of uveitis can lead to irreversible ocular damage and result in significant visual impairment [4, 7]. The first two common causes of moderate and severe visual loss in uveitis are chronic cystoid macular edema and macular scarring respectively [8]. Factors associated with visual loss in uveitis have been previously studied. In HLA B-27-associated uveitis, the presence of posterior synechiae, corticosteroid-sparing therapy, corticosteroid injections, chronic diseases, and male gender were associated with vision loss (20/50 or worse) [9]. To our knowledge, there have been no studies reporting factors associated with visual outcome in uveitis cases in Thailand. The present study aims to address the following clinical research questions:

- What is the pattern of uveitis in the tertiary center of Thailand?
- What are the factors associated with poor visual outcome in uveitis patients in the tertiary center of Thailand?

## Materials and methods

This is an ongoing prospective, single-center, prospective cohort study that will include patients seen at King Chulalongkorn Memorial Hospital (KCMH), Thailand between September 1, 2022, to December 31, 2027. This study was reviewed and approved by the Ethics Committee of the Faculty of Medicine, Chulalongkorn University (REC no. 0784/2022). Written informed consent will be obtained from all subjects for prospective data collection. The patient data is maintained confidentiality. The study adheres to the Declaration of Helsinki.

### Target population

We are targeting a total of 500 adult patients who presented to the uveitis clinic at King Chulalongkorn Memorial Hospital (a major referral center in the central region of Thailand) between September 1, 2022 and December 31, 2027.

#### Inclusion criteria

Patients diagnosed as having uveitic diseases and able to follow up at least 1 year from baseline.

#### Exclusion criteria

Patients who have traumatic or surgical-related ocular inflammation.

### Trial design and follow-up

At the first visit, the patient’s demographic information is reviewed. Additional data related to the onset, course, duration, previous medications, and current treatment of uveitis are noted. The best-corrected visual acuity (BCVA), slit-lamp examination, applanation tonometry, fundus examination, and indirect ophthalmoscopy are recorded at each visit. Ancillary ocular imaging and laboratory testing including optical coherence tomography (OCT), B-scan ultrasonography, fundus fluorescein angiography, computed tomography (CT) of chest scans, plain X-ray sacroiliac joints, and/or magnetic resonance imaging (MRI), complete blood count with sedimentation rate, serum angiotensin-converting enzyme (ACE), purified protein derivative (PPD) skin test, syphilis serology (VDRL, TPHA); toxoplasma serology, HLA-B27 typing, ELISA test for human immunodeficiency virus (HIV), and QuantiFERON TB gold are also recorded. Finally, the factors associated with poor visual outcome are reviewed including etiology of uveitis, age, sex, diagnosis, medication history, history of steroid use, smoking history, systemic co-morbidities, the presence of cataract, surgeries performed, ocular complications including the development of cataract, epiretinal membranes, retinal neovascularization, posterior synechiae, intraocular hemorrhage, ocular hypertension, glaucoma, and central macular thickness automatically calculated by Swept Source OCT (Triton TM, TOPCON, Japan).

The activity grading and type of intraocular inflammation (uveitis) are classified according to The Standardization of Uveitis Nomenclature (SUN) working group [2]. Uveitis is considered active if any of the following signs are present: anterior chamber cells ≥1+, vitreous haze ≥1+, and the presence of active retinitis or choroiditis [2]. The BCVA results were converted to logMAR units for statistical analysis. Mild or no visual loss (MdVL) is defined as >20/50 on Snellen chart (logMAR <0.4). Moderate visual loss (MVL) is defined as 20/50–20/200 (logMAR 0.4 to <1.0). Severe visual loss (SVL) is defined as 20/200–20/400 (logMAR ≥1.0). Blindness is defined as <20/400 (logMAR ≥1.3) [10]. Poor visual prognosis is defined as having moderate or severe visual loss at a 1-year follow-up.

The follow-up interval varies depending on the severity of each patient. Patients whose medications needed to be adjusted or who have active diseases may follow up weekly, whereas patients with stable and inactive diseases may follow up every 4 to 8 weeks.

### Safety

Since this study is observational, there are no direct risks associated with participation.

### Statistical considerations

#### Sample size estimation

We planned to screen all patients presenting at our department for uveitis observation or treatment. We aim to recruit 500 cases with uveitis diagnoses.

#### Statistical analysis

The prevalence of uveitis according to anatomical types and specific diseases will be examined at the time of diagnosis. The factor associated with loss of visual acuity in uveitis patients will be evaluated by multivariate analysis. Continuous variables will be statistically evaluated using the Mann–Whitney U test, while categorical variables will be analyzed using the chi-square test.

## Discussion

Uveitis is responsible for up to 20% of all cases of legal blindness in developed countries as well as 25% of blindness in developing countries [11]. It might cause a life-long burden to the patient in many aspects including quality of life, potential side effects associated with local and systemic immunosuppressive medications, and the cost of treatment [12]. A better understanding of the pattern and factors associated with visual outcomes for uveitic patients is therefore required for the treatment plan.

CU^2^C study is a large, prospective cohort study for all uveitis presented at King Chulalongkorn Memorial Hospital, Thailand between September 1, 2022, to December 31, 2027. This study is designed as a 5-year, longitudinal follow-up investigating the prevalence and visual prognosis of uveitis patients. Although the pattern of uveitis in Thai patients among different regions in Thailand has been previously described [3, 5, 6], the factor associated with poor visual acuity in uveitis patients remains unknown. In addition, the previous studies in Thailand have some limitations. Certain laboratory investigations such as polymerase chain reaction for viral multiplex, QuantiFERON-TB Gold, and toxoplasmosis serologic testing were not available. This could result in an excessively high number of cases of idiopathic uveitis. In King Chulalongkorn Memorial Hospital, several different laboratory investigations are now available. Therefore, it is likely to reveal a more complete spectrum of diagnoses compared to previous studies. The major limitation of this study is a referral bias as the clinical characteristics and frequency of patients presenting to tertiary-care referral centers may be different from those in the community or general population. Therefore, clinicians should be aware of the consequences of this bias before extrapolating data to other settings.

### Conclusions

This study will reveal the prevalence and the factor associated with poor visual acuity of uveitis in Thai patients.
